# Drying of a plasmid containing formulation: chitosan as a protecting agent

**DOI:** 10.1186/2008-2231-20-22

**Published:** 2012-09-03

**Authors:** Nasir Mohajel, Abdolhossein R Najafabadi, Kayhan Azadmanesh, Mohsen Amini, Alireza Vatanara, Esmail Moazeni, Amirabbas Rahimi, Kambiz Gilani

**Affiliations:** 1 Aerosol Research Laboratory, Department of Pharmaceutics, School of Pharmacy, Tehran University of Medical Sciences, Tehran, Iran; 2 Department of Pharmaceutics, Faculty of Pharmacy, Shahid Sadoughi University of Medical Sciences, Yazd, Iran; 3 Department of Virology, Pasteur Institution of Iran, Tehran, Iran; 4 Department of Medicinal Chemistry, School of Pharmacy, Tehran University of Medical Science, Tehran, Iran

**Keywords:** Nanocomplex, Polymeric gene delivery, Gene therapy, Spray-drying, Freeze-drying

## Abstract

**Background:**

Along with research on development of more efficient gene delivery systems, it is necessary to search on stabilization processes to extend their active life span. Chitosan is a nontoxic, biocompatible and available gene delivery carrier. The aim of this study was to assess the ability of this polymer to preserve transfection efficiency during spray-drying and a modified freeze-drying process in the presence of commonly used excipients.

**Methods:**

Molecular weight of chitosan was reduced by a chemical reaction and achieved low molecular weight chitosan (LMWC) was complexed with pDNA. Obtained nanocomplex suspensions were diluted by solutions of lactose and leucine, and these formulations were spray dried or freeze dried using a modified technique. Size, polydispersity index, zeta potential, intensity of supercoiled DNA band on gel electrophoresis, and transfection efficiency of reconstituted nanocomplexes were compared with freshly prepared ones.

**Results and conclusion:**

Size distribution profiles of both freeze dried, and 13 out of 16 spray-dried nanocomplexes remained identical to freshly prepared ones. LMWC protected up to 100% of supercoiled structure of pDNA in both processes, although DNA degradation was higher in spray-drying of the nanocomplexes prepared with low N/P ratios. Both techniques preserved transfection efficiency similarly even in lower N/P ratios, where supercoiled DNA content of spray dried formulations was lower than freeze-dried ones. Leucine did not show a significant effect on properties of the processed nanocomplexes. It can be concluded that LMWC can protect DNA structure and transfection efficiency in both processes even in the presence of leucine.

## Introduction

While viral gene delivery systems are more efficient and targeted than non-viral gene delivery systems in transducing cells, there are serious concerns about their safety [1]. In addition, they have limited capacity for DNA packing and are not easy to produce. These major shortcomings have become a motive force for research on safe and efficient non-viral gene delivery systems. Polymers have received more attention because their ease of production and modification can be applied to overcome major defects of non-viral gene delivery systems such as low transfection, targeting efficiency and toxicity [2].

Alongside efforts to make more safe and efficient gene carriers, it is necessary to make them stable during storage. Stability of non-viral gene delivery systems has been a major concern since the first days of gene therapy clinical trials [3]. Presence of water can alter the stability of non-viral gene delivery systems in short term storage by facilitating their aggregation [4] and in long term by increasing the likelihood of water catalyzed chemical reactions, which may alter the stability of nucleic acids [5]. On the other hand, low efficiency of non-viral gene delivery systems calls for application of high doses of DNA in a limited volume *in-vivo*, which cannot be achieved during preparation of polymeric gene delivery systems. Because polyplexes aggregate in high concentrations [6]. Removing water from polyplex suspensions, results in pharmaceutically reasonable stability profile at room temperature and simultaneously increases concentration of therapeutic gene in the formulation. These formulations can be applied as dry powder (in respiratory gene delivery) or reconstituted with water before application.

In solid state, polymer based gene delivery systems have an advantage over lipid based ones because they are less vulnerable to oxidative reactions [7]. Although freeze-drying is a well-established industrial approach, conventional freeze-drying can exacerbate aggregation in its freezing step by concentrating nanosuspension in unfrozen phase. Therefore, special modifications have been considered to increase cooling rate to overcome this defect [8]. On the other hand, it has been shown that spray-drying of insulin loaded chitosan/tripolyphosphate nanoparticles did not change their size and zeta potential significantly [9]. This was attributed to the high drying speed [10]. However, it has been reported that excessive shear rate can destroy supercoiled structure of plasmid [11]. Appropriate design of a gene delivery system is of critical importance not only for protecting nucleic acids *in-vivo* but also for preserving them against the process stresses like temperature and shear.

The ability of a lipid-protein gene delivery system to protect DNA in a spray-drying process and the effects of various excipients on transfection efficiency and aerodynamic properties of the resulted powder had been thoroughly studied by Li *et al. *[12,13]. *In vivo* gene transfer performance of a chitosan-pDNA complex processed by supercritical technology have been studied by Okamuto *et al.*[14]. Regarding DNA stability in supercritical fluid technology, the main destabilizing factor is dissolved carbon dioxide in water, which builds up an acidic pH and promotes DNA hydrolysis, while in spray-drying, the shear stress and temperature are main destabilizers.

Availability, biocompatibility, inherent positive charge and permeability enhancing capability make chitosan an attractive non-viral gene delivery system [15]. Polyion complex associates of chitosan and DNA are strong and have been suggested to be further stabilized by hydrogen and hydrophobic bonds. It has also been hypothesized that these bonds are strong enough to prevent DNA liberation from complexes and thus decrease free plasmid content after endosomal escape and detract from transfection efficiency of the polymer [16]. Molecular weight reduction has been applied as a successful strategy to increase the transfection efficiency of chitosan, with a suggested mechanism of loosening the bond between DNA and the polymer [17]. So, Chitosan can be applied as a good representative to study protection of plasmid DNA by polymeric gene delivery systems against the process stresses.

The aim of this study was to evaluate the ability of low molecular weight chitosan to protect bioactivity of pDNA against shear and thermal stresses generated during spray-drying and high freezing rate freeze-drying processes and study the effect of leucine as a commonly used excipient in preparation of spray dried formulations on this protection.

## Materials and methods

### Materials

Medium viscose chitosan was obtained from Primex (Siglufjordur, Iceland). Sodium nitrite, sodium hydroxide, glacial acetic acid and L-leucine were purchased from Merck KGaA (Frankfurt, Germany). Lactose monohydrate free sample was provided by DMV-Fonterra Excipients (Foxhol, Netherlands). All cell culture materials were obtained from Invitrogen (CA, USA).

### Amplification of plasmid

pEGFP-N1 plasmid (Clontech Laboratories, CA, USA) encoding the enhanced green fluorescent protein as a reporter was amplified in *Escherichia coli* DH5α by overnight incubation of the bacteria and then purification of the plasmid by a Giga plasmid purification kit (Nucleobond^TM^ PC 10000, Macherey-Nagel GmbH & CO.KG, Düren, Germany). The resulted plasmid was analyzed for its purity and concentration by gel electrophoresis and UV absorption at 260 and 280 nm (Picodrop^TM^, Saffron Walden, UK).

### Preparation of low molecular weight chitosan (LMWC)

Low molecular weight chitosan was prepared by chemical reaction between chitosan and nitrous acid. Two grams of medium viscose chitosan (Primexehf, Siglufjordur, Iceland) slowly dispersed into 78 ml deionized water. Then, 6 ml glassial acetic acid was added to the dispersion to dissolve chitosan. Nitrogen bubbled into this solution and then 16 ml of a freshly prepared sodium nitrite solution with a concentration of 5 mg/ml was added. Reaction mixture was sealed by a cap and stirred in room temperature for 90 minutes then pH was adjusted to 9 by adding 4 M sodium hydroxide to terminate the reaction. The resulting suspension was centrifuged at 6000 rpm, and the sediment was washed by ice-cold water thoroughly over a buchner funnel, redissolved in a minimum amount of 1% acetic acid solution and dialyzed against two liters of deionized water, which was replaced every 12 hours, for 48 hours. The resulted solution was freeze-dried, and the powder was kept at 2-8°C in the dark until use.

Molecular weight (M_w_, M_n_) and polydispersity (M_w_/M_n_) of LMWC were determined by gel permeation chromatography. The polymer dissolved at the concentration of 0.1% in 0.3 M acetate buffer with a pH value of 4.6. The resulted solution filtered through a 0.22 μ filter and chromatographed at 25°C using the same eluent at a flow rate of 4 ml/min through a PL aquagel-OH Mixed –H 25 mm ID 8 μm column on a Knuer chromatographic system (Knuer, Berlin, Germany), which was equipped with an intrinsic viscosity detector. Pullulan standards in five molecular weights were used for calibration using the same conditions.

### Particle formation

To prepare a stock solution of LMWC, the polymer was dissolved in 0.005 M acetate buffer with a pH value of 5 and its concentration was adjusted to 7 mg/ml, then this solution was centrifuged at 30000 Relative Centrifugal Force (RCF) for 60 minutes in 4°C (Sigma Laborzentrifugen GmbH, Osterode, Germany) and the supernatant was filtered through a 0.22 μ filter. This solution was stored at 2-8°C in the dark and used within a week from the date of preparation.

Nanocomplexes of LMWC and pEGFP-N1 were prepared using the ionic gelation method. For preparation of each nanocomplex formulation predetermined volumes of LMWC stock solution were diluted with the same solvent to obtain target concentration and was added to the same volume of pDNA solution with concentration of 40 ng/μl, which was prepared in Milli-Q water, in three increments at room temperature and under the vortex. After addition of LMWC to pDNA solution, the mixture was incubated in room temperature for 20 minutes for the particle formation before further experiments.

### Spray-drying

Nanocomplex formulations spray dried immediately after formation (Buchi B-191 mini-spray dryerTM; Buchi Labortechnik AG, Switzerland) to make powder formulations given in Table 1. The spray-drying conditions were the same as reported by Li *et al.*[13], which were: inlet temperature 150°C, spray flow rate 600 liter/h, aspirator adjusted to 35 m3/h, pump adjusted to 30% to give a flow rate of 7.5 ml/min. These conditions resulted in an outlet temperature of 70-85°C. The spray-drying yield was calculated, and the recovered powders stored in moist resistant containers at 2-8°C until next experiments.

**Table 1:** Theoretical composition of freeze dried and spray dried powders

**Formulations**																
**Components**	**100 L**	**100 N**	**70 L**	**70 N**	**50 L**	**50 N**	**20 L**	**20 N**	**10 L**	**10 N**	**7 L**	**7 N**	**5 L**	**5 N**	**2 L**	**2 N**
DNA	0.5	0.5	0.5	0.5	0.5	0.5	0.5	0.5	0.5	0.5	0.5	0.5	0.5	0.5	0.5	0.5
LMWC	33.7	33.7	23.6	23.6	16.8	16.8	6.7	6.7	3.4	3.4	2.36	2.36	1.68	1.68	0.67	0.67
Lactose	2000	2000	2000	2000	2000	2000	2000	2000	2000	2000	2000	2000	2000	2000	2000	2000
Leucine	-	200	-	200	-	200	-	200	-	200	-	200	-	200	-	200

All measurements are in milligrams.

### Freeze-drying

Nanocomplex formulations were freeze-dried immediately after preparation by a modified freeze-drying technique. Briefly, prepared nanosuspensions were transferred to cryotubes and immersed in liquid nitrogen for 10 minutes to snap freeze the nanosuspensions, then these tubes were transferred to a pre-cooled freeze-dryer chamber (Alpha 1-4 LD plusTM, Martin Christ Gefriertrocknungsanlagen GmbH, Osterode, Germany) after loosening their caps. Freeze-drying conditions were: 48 h in 0.04 mbar to give an ice condenser temperature of -50°C and 12 h in 0.001 mbar for ice condenser temperature of -80°C.

### Particle size and zeta potential analyses

Particle size and zeta potential of the nanocomplexes were measured before and after freeze or spray-drying by a Zetasizer Nano ZS^TM^ (Malvern Instruments, Malvern, Worcestershire, UK). Freshly prepared nanocomplexes and dry powders were diluted in Milli-Q water to give a count rate of 100-200 KC/S. All measurements were performed at 25°C in triplicate. Viscosity and refractive index of water in this temperature (0.8905 mPa s, 1.333) were used for data analysis.

### Stability of pDNA

To analyze the integrity of DNA after spray and freeze-drying, plasmid DNA was electrophoresed after dissociation from LMWC. To dissociate DNA, an amount of each nanocomplex formulation equivalent to 1.5 μg of DNA was dispersed in 30 μl water. This suspension was added to 1.5 mg of heparin, which was dissolved in the same volume of water and incubated for five hours at room temperature. After the incubation time, 20 μl of the resulted suspension was mixed with 4 μl of loading dye and was loaded into each well of a 0.5% agarose gel, which were stained with EtBr, and electrophoresed at a voltage of 100 V, in TBE buffer (pH 8) for 60 minutes. Gel images were acquired by a BioDoc-ItTM imaging system (UVP, Upland, CA, USA), while uncomplexed pDNA was used as control. The acquired images were processed to reduce EtBr background fluorescence and analyzed by Image J software (Rasband, W.S., ImageJ, U. S. National Institutes of Health, Bethesda, Maryland, USA, http://rsb.info.nih.gov/ij/, 1997-2009).

### In-vitro transfections

The day before transfection, each well of a 24 well cell culture plate, containing 1 ml of DMEM media supplemented with 10% FBS and 1% penicillin-streptomycin, was seeded with 70000 low passage 293 T cells. Transfection was conducted when confluency was about 80%. Before transfection, the media was replaced with 500 μl of fresh serum free media (at pH 6.9). After replacement of media 100 μl of nanocomplex formulation or reconstituted powder (equal to 2 μg of pEGFP-N1) were transferred to each well. Cells were incubated at 37°C, 5% CO2, with nanocomplexes for 4 h and then the medium replaced by 1 ml of fresh serum containing media.

Seventy-two hours later, cells were washed with warm PBS buffer twice and trypsinized for 5 minutes. Then 500 μL PBS buffer, supplemented with 10% FBS was added to neutralize trypsin and cells were analyzed by flow cytometry (CyFlow^TM^, Partec GmbH, Münster, Germany) to assess GFP expression.

### Statistical analysis

To compare particle size distribution curves two different techniques were applied,(i.e. Kolmogorov-Smirnov (K-S test) and similarity factor (f_2_)) K-S test was selected as a non-parametric method to compare distribution curves of the processed nanocomplexes by freshly prepared ones. Similarity factors were calculated using the following equation as a parallel test:

(1) f2=50×log1+1n∑j=1nwj|Rj−Tj|−05×100

In which R_j_ and T_j were the_ average intensity percent given by the instrument for a particular size (j) and wj is a weight factor for each size point, which here was set to be 1. These tests were selected because in the case of particle size the assumption for parametric tests (i.e. *t*-test) that sample is normally distributed is not correct and these distribution profiles are mostly right-skewed and bimodal, which cannot be fully represented by mean and standard deviation.

To study the effect of process, addition of leucine and N/P ratio on stability of pDNA and transfection efficiency a three-way analysis of variance was used.

## Results

### Molecular weight

Chemical reaction with NaNO_2_ has been used before for depolymerization of chitosan. The molecular weight of the polymer was demonstrated to reduce linearly with the ratio of chitosan/NaNO_2_[18]. The molecular weight of chitosan was determined by gel permeation chromatography coupled with refractive index detector. The number and weight molecular weight of LMWC were 9.8 kDa and 16 kDa respectively, which resulted in a polydispersity index of 1.63.

### Size and zeta potential of nanoparticles

**Figure 1:**
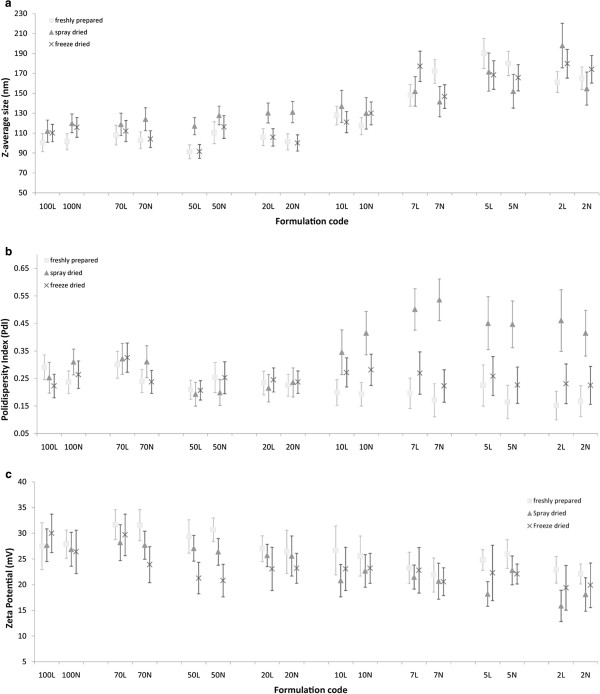
**Size, polydispersity and zeta potential of freshly prepared and reconstituted nanocomplexes from freeze dried or spray dried powders. a**, z-average size, **b**, polydispersity index and **c**, zeta potential, Points are averages of three measurements and error bars are standard deviation.

Size, polydispersity index (PdI) and zeta potential data of the nanocomplex formulations before and after processing by spray-drying or freeze-drying are presented in Figure 1. Reduction of N/P ratio increased the Z-average particle size and polydispersity index and decreased the zeta potential.

To determine success of the two processing techniques in preservation of particle size of nanocomplexes, Kolmogorov-Smirnov (K-S test) test, a nonparametric statistical test, was used. Similarity factor was also used to compare average particle size distribution curves of the freshly prepared nanocomplex formulations with processed ones. Results of these two tests are presented in Tables 2 and 3. The *p*-Values for K-S test (Table 2), showed that all formulations prepared by freeze-drying and 13 out of 16 spray-dried formulations successfully preserved the original size distribution profile of nanocomplexes over a broad range of N/P ratios. Results of K-S test for particle size comparison were in consistent with polydispersity data (Figure 1b). Calculated similarity factors showed a reduction as N/P ratio decreased and the statistical difference between formulations became significant when the similarity factor was below 82.

**Table 2:** ***p- *Values of K-S tests for comparison of size distribution profiles of processed nanocomplexes with freshly prepared ones**

**Formulations**																
**100 L**	**100 N**	**70 L**	**70 N**	**50 L**	**50 N**	**20 L**	**20 N**	**10 L**	**10 N**	**7 L**	**7 N**	**5 L**	**5 N**	**2 L**	**2 N**	
Freeze dried	0.860	0.508	0.822	0.713	0.943	0.877	0.84	0.806	0.205	0.08	0.109	0.518	0.681	0.448	0.244	0.595
Spray dried	0.84	0.777	0.240	0.38	0.748	0.174	0.799	0.79	0.115	0.167	0.799	0.343	0.392	0.02^*^	0.02^*^	0.045^*^

^*^statistically meaningful difference from original size distribution profile.

**Table 3:** Calculated similarity factors between size distribution profiles of freeze dried and spray dried nanocomplexes with those of freshly prepared ones

**Formulations**																
	**100 L**	**100 N**	**70 L**	**70 N**	**50 L**	**50 N**	**20 L**	**20 N**	**10 L**	**10 N**	**7 L**	**7 N**	**5 L**	**5 N**	**2 L**	**2 N**
Freeze dried	93.73	93.13	97.31	97.39	94.36	98.79	97.5	99.35	90.96	87.32	88.48	83.19	87.49	86.15	93.67	90.31
Spray dried	95.84	94.41	95.14	89.33	88.93	86.85	89.56	90.38	85.62	83.79	94.25	93.35	86.57	81.24	74.41	81.61

The data indicated that the average zeta potential decreased from around 30 for N/P equal to 100 and 70 to around 20 for N/P of 5 and 2.

Comparing the processed nanocomplexes with similar N/P ratios, the K-S test did not show significant differences between particle size distribution curves of the formulations prepared in presence or absence of leucine (all *p-*values were above 0.05).

### Stability of pDNA

**Figure 2:**
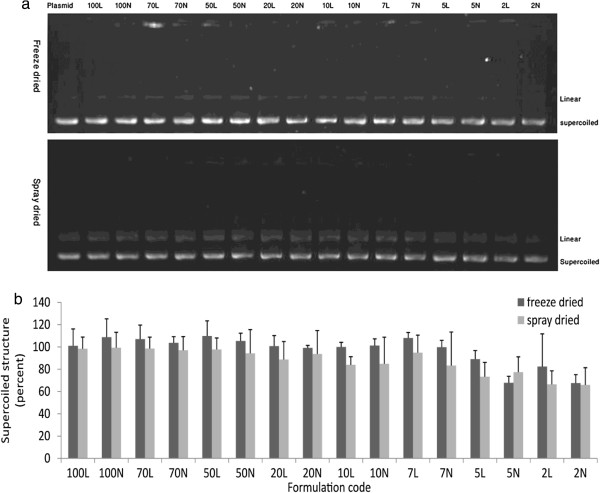
**a, Electrophoresis images of dissociated pDNA from reconstituted nanocomplexes processed by freeze-drying (upper) and spray-drying (lower) pure pDNA was used as control. b**, relative intensity of supercoiled pDNA band of freeze dried and spray dried nanocomplexes. Error bars are standard deviation (n = 3).

Stability of plasmid bounded with LMWC after freeze-drying and spray-drying processes was determined by electrophoresis. Figure 2a, shows images of two sample gels and Figure 2b presents percent of preserved supercoiled structure in the both processes. Nanocomplex formulations prepared with N/P ratios of 2 and 5 showed lowest stability of DNA during both processes, with approximately 70 percent of the supercoiled pDNA remaining intact.

A three-way ANOVA test used to study the effect of process, addition of leucine and N/P ratio on supercoiled structure stability. There was a significant difference between the effect of freeze-drying and spray-drying processes on preserving the supercoiled structure (*p* <0.001). In addition, it was shown that the stability of supercoiled structure was increased significantly with the increase in N/P ratio (*p* < 0.001). The test did not show a significant destabilization effect for leucine on supercoiled structure (*p* = 0.184).

### In-vitro transfection

**Figure 3:**
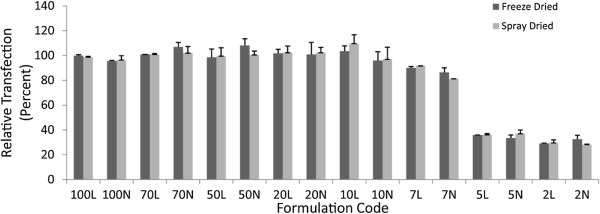
**Relative transfection efficiency of reconstituted freeze dried or spray dried powders. **Error bars are standard deviation (n = 3).

The relative transfection efficiency of the nanocomplexes is presented in Figure 3. Except for N/P ratios of 2 and 5, all N/P ratios in both kinds of processed powders could retain approximately their original transfection efficiency. Even spray dried powders showed 100% relative transfection, although their supercoiled pDNA content was lower than the freeze-dried powders. To analyze the effect of the process, the addition of leucine and the N/P ratios a three-way analysis of variance was conducted. The test did not show a meaningful effect for the type of process or addition of leucine (*p* = 0.827and *p* = 0.476, respectively), while the effect of N/P ratio was reemphasized(p < 0.001).

## Discussion

Effects of three different variables (i.e. processing technique, N/P ratio and presence of leucine) on physicochemical and biological properties of nanocomplexes were tested by statistical analysis. These effects have been discussed below.

### Effect of N/P ratio

The decrease in the number of positively charged amine groups with respect to negatively charged phosphate groups led to a reduction in zeta potential and in turn, made the nanocomplexes more vulnerable to aggregate. It has been shown that chitosan-DNA nanocomplexes aggregate quickly in Hank’s buffered salt solution at the amine per phosphate ratio of 5 [19]. Increased aggregation rate in lower N/P ratios makes stabilization of the nanocomplexes more challenging [20]. This effect can be seen in the difference of particle size distribution of three spray-dried formulations with lower N/P ratios form freshly prepared ones.

According to our results N/P ratio had a significant effect on stability of supercoiled structure and preservation of biological activity during both tested processes. It has also been demonstrated in previous researches that increased N/P ratio can increase physical stability of DNA containing nanocomplexes against shear stress [21]. The level of protection also depends on the type of complexing agent. Lipoplexes are shown to be more vulnerable against shear stress generated during nebulization (27).

### Effect of processing technique

Kuo *et al.*[21] compared Z-average particle sizes of poly(ethyleneimine)-pDNA polyplexes before and after a spray-freeze-drying process and observed no statistically meaningful difference between the two groups. However, to our knowledge there are not more reports, comparing size distribution of nanocomplexes prepared by pDNA and a polymeric carrier before and after a drying process.

The high rate of aggregation of LMWC-pDNA nanocomplexes necessitates instant immobilization of the system to prevent changes in particle size. Although both techniques applied in this work have such a capability, but spray-drying of liquid formulations took more time than freezing in liquid nitrogen to immobilize the nanocomplexes. Therefore, it seemed that in low N/P ratios, the rate of aggregation of the nanocomplexes was higher than the rate of drying of the nanocomplexes during the spray-drying process.

Stability of the supercoiled structure can also be altered by processing technique. Seville *et al.* freeze dried a lipid :polycation :pDNA vector with a similar freeze-drying technique [11]. Their results showed 100% stability of supercoiled DNA during the process. Mohri *et al.*, showed that chitosan can protect pDNA in N/P ratios over 5 during a spray freeze-drying process [22]. It was shown that a freeze drying process with increased cooling rate can improve preservation of biological activity of non-viral gene delivery systems [8], despite the fact that rapid freezing of naked DNA can extensively damage its structure [23,24]. Our results also indicated that by applying a similar freeze-drying condition, in N/P ratios > 10, a 100% preservation of supercoiled plasmid could be obtained.

On the other hand, spray-drying is known to be a harsher technique compared to freeze-drying. Seville *et al.* recovered 70-80% of original supercoil band strength after processing of a lipid-protein carrier by spray-drying technique [13], but here we achieved over 90% of the band strength in N/P ratios of 10, 20 and 50 and even 100% at N/P ratios of 70 and 100.

While powders processed by spray-drying showed lower stability profile in nanocomplex size and supercoiled DNA content, compared to freeze-dried ones, their transfection efficiencies were not significantly affected. Our data is in accordance with Seville and coworkers, who has reported an enhancement in transfection efficiency of spray dried powders despite lower supercoiled DNA content compared to freeze dried formulations. They did not compare size distribution of nanocomplexes before, and after processing but our data showed an increase in polydispersity of spray-dried formulations, which along with reduction in content of supercoiled structure should have led to a decrease in biological activity of the formulations. These findings suggest even enhancement of transfection activity in spray-drying or reduction of this ability in freeze-drying via unknown mechanisms.

There are some reports indicating enhanced gene expression after freeze-drying or spray-drying of gene delivery systems. Seville *et al.* witnessed a 50% increase in gene expression using powders processed by spray-drying technique but freeze-dried powders did not show the same result [11]. Talsma *et al.* observed enhanced transfection efficiency after freeze-drying of an adenovirus-enhanced AVET system, but in the case of pCMVL:transferrin-poly(ethlenimine) complexes, they showed 100% of original transfection efficiency after freeze-drying in a 10% sucrose solution [25]. Furthermore, Kuo and Hwang did not report an enhancement in transfection ability of poly(ethyleneimine)-DNA complexes processed by spray-freeze-drying [21]. In this work, we did not see a meaningful increase in transfection efficiency after spay drying and freeze-drying.

### Effect of leucine

Leucine is a common excipient in the formulation of dry powder inhalers to enhance dispersibility of the powders. A previous report indicated that co-processing of a lipid-polycation-pDNA with leucine and lactose led to the reduction of the biological activity of the plasmid, compared to those which formulated with just lactose in transfecting A549 cells [26]. Therefore, it has been suggested that leucine acts as a destabilizer for complex between DNA and a positively charged material. We added leucine to our powder formulations to assess the ability of chitosan in maintaining original size distribution, DNA structure and transfection in the presence of this excipient. Our results did not show a meaningful effect for leucine on any of the mentioned indicators. This effect can be attributed to higher resistance of chitosan bond with DNA against the effect of leucine. Köping-Höggård and coworkers found that incubation with sodium chloride, 3.5 M, sodium dodecyl sulfate, 0.5 M or heparin, with a 10-fold excess of negative charges could not liberate DNA from its complex by ultrapure chitosan. Based on these works higher stability of chitosan nanocomplexes with plasmid is plausible.

## Conclusion

In this work, we tried to assess the ability of LMWC as a carrier to protect supercoiled structure and thus transfection ability of plasmid DNA, and maintain the original size of the freshly formed nanocomplexes during the harsh conditions of spray-drying and freeze-drying processes. Our indicators were size, zeta potential, polydispersity index, intensity of supercoiled bond on electrophoresis gel and transfection efficiency in in-vitro conditions.

LMWC could efficiently protect plasmid supercoiled structure and retain its transfection capability against stresses imposed by freeze-drying and spray-drying processes. The stability of supercoiled structure was also retained in the presence of leucine, which has been reported to destabilize the complex between DNA and a positively charged material. Our finding suggests that LMWC carrier is resistant to the drying process stresses imposed to gene delivery systems.

## Author’s contribution

*Concept and design*: KG, NM, KA, MA. *Acquisition of data*: NM, EM, AR. *Analysis and interpretation of data*: NM, KG, KA, AV, ARN. *Drafting and revising*: NM, KG, KA, ARN. All authors read and approved the final manuscript.
